# A neuroligin-3 mutation implicated in autism causes abnormal aggression and increases repetitive behavior in mice

**DOI:** 10.1186/s13229-015-0055-7

**Published:** 2015-11-14

**Authors:** Emma L. Burrows, Liliana Laskaris, Lynn Koyama, Leonid Churilov, Joel C. Bornstein, Elisa L. Hill-Yardin, Anthony J. Hannan

**Affiliations:** Florey Institute of Neuroscience and Mental Health, The University of Melbourne, Kenneth Myer Building, Melbourne Brain Centre, Cnr Genetics Lane and Royal Pde, Parkville, Victoria 3010 Australia; Florey Institute of Neuroscience and Mental Health, 245 Burgundy St, Heidelberg, Victoria 3084 Australia; Department of Physiology, The University of Melbourne, Royal Pde, Parkville, Victoria 3010 Australia; Department of Anatomy and Neuroscience, The University of Melbourne, Royal Pde, Parkville, Victoria 3010 Australia

**Keywords:** Autism, Brain disorder, Cognitive disorder, Gene mutation, Mutant mice, Neuroligin-3, Psychiatric disorder, Repetitive and restrictive behavior, Social interaction, Synaptic protein

## Abstract

**Background:**

Aggression is common in patients with autism spectrum disorders (ASD) along with the core symptoms of impairments in social communication and repetitive behavior. Risperidone, an atypical antipsychotic, is widely used to treat aggression in ASD. In order to understand the neurobiological underpinnings of these challenging behaviors, a thorough characterisation of behavioral endophenotypes in animal models is required.

**Methods:**

We investigated aggression in mice containing the ASD-associated R451C (arginine to cysteine residue 451 substitution) mutation in neuroligin-3 (NL3). Furthermore, we sought to verify social interaction impairments and assess olfaction, anxiety, and repetitive and restrictive behavior in NL3^R451C^ mutant mice.

**Results:**

We show a pronounced elevation in aggressive behavior in NL3^R451C^ mutant mice. Treatment with risperidone reduced this aggression to wild-type (WT) levels. Juvenile and adult social interactions were also investigated, and subtle differences in initiation of interaction were seen in juvenile NL3^R451C^ mice. No genotype differences in olfactory discrimination or anxiety were observed indicating that aggression was not dependent on altered olfaction, stress response, or social preference. We also describe repetitive behavior in NL3^R451C^ mice as assessed by a clinically relevant object exploration task.

**Conclusions:**

The presence of aberrant aggression and other behavioral phenotypes in NL3^R451C^ mice consistent with clinical traits strengthen face validity of this model of ASD. Furthermore, we demonstrate predictive validity in this model through the reversal of the aggressive phenotype with risperidone. This is the first demonstration that risperidone can ameliorate aggression in an animal model of ASD and will inform mechanistic and therapeutic research into the neurobiology underlying abnormal behaviors in ASD.

**Electronic supplementary material:**

The online version of this article (doi:10.1186/s13229-015-0055-7) contains supplementary material, which is available to authorized users.

## Background

Autism spectrum disorder (ASD) is a behaviorally defined condition involving a broad range and degree of symptom expression for which the etiology is unknown [1]. The study of highly penetrant ASD susceptibility genes have identified convergent downstream pathways involved in ASD [2, 3]. Furthermore, systems biology approaches have combined disease risk, protein-protein interaction, gene ontology, conserved domains, and transcriptomics databases to create functional networks of genes implicated in ASD. The results of these studies group genes in functional networks enriched for processes such as neuronal migration, cytoskeleton dynamics, axon growth, and importantly, trans-synaptic signaling as a major network hub [4, 5].

Neuroligins are synaptic cell-adhesion molecules that mediate trans-synaptic signaling through their binding partner neurexins and shape neural network properties by specifying synaptic functions [6]. Importantly, mutations in genes encoding neuroligins [7] and neurexins [8] have been identified in ASD patients. To study the mechanisms by which these mutations and subsequent disruptions to cellular processes contribute to ASD, animal models have been generated that recapitulate important aspects of the human disorder. These animal models have become a major *in vivo* tool to investigate the neurobiological mechanisms leading to the expression of behavioral traits in ASD.

Mice expressing the arginine to cysteine residue 451 substitution (R451C) mutation in the synaptic adhesion gene neuroligin-3 (NL3) have been proposed as a model of ASD. The ASD-causing point mutation in neuroligin-3 causing R451C substitution [7] impairs intracellular trafficking and reduces neuroligin-3 protein levels at the cell membrane by 90 % [9, 10]. NL3^R451C^ mice show a diverse range of behavioral abnormalities and synaptic dysfunction as shown by increased cortical inhibition together with enhanced hippocampal excitation in brain slices [9, 11–14]. However, conflicting reports have arisen from studies of social behavior in NL3^R451C^ mice [9, 15].

While the core diagnostic symptoms include social communication deficits alongside the presence of repetitive behaviors, patients also exhibit a variety of comorbid traits such as aggression, which adversely impact on patient quality of life [16]. Aggression is observed in up to 70 % of individuals with ASD [17] and significantly limits patient access to education, health care, and employment [18]. The atypical antipsychotic risperidone is frequently prescribed for treatment of aggressive behaviors in children with ASD [19]; however, side effects significantly limit its use [20–22]. The serious negative outcomes associated with behavioral impairments in ASD highlight the inherent importance of understanding the underlying neurobiological mechanisms with the aim of developing more effective treatments.

Aggression in mice is a robust, innate, social behavior and serves to assist the acquisition of social ranking and resources from the environment. Dominance hierarchies are established and maintained through confrontations between rival male mice. Territorial aggression between males can be measured utilising the resident-intruder test whereby a juvenile intruder mouse is introduced to the home cage of a test mouse [23]. This paradigm mimics many elements of the behavior displayed by resident males to exclude other breeding males from their home territory and their mates [23]. Escalated, pathological, and abnormal forms of aggression are characterised by prolonged and frequent attacks and brief latencies to attack following the introduction of an intruder mouse [24, 25].

In order to determine whether the NL3 R451C mutation impacts on social and repetitive behavior in mice, we assessed aggressive interactions in this model. Juvenile social interaction and adult sociability were also investigated in an attempt to resolve previously conflicting reports in the literature [9, 11, 13, 15]. Repetitive and restrictive behavior was also investigated using an adaptation of a clinical task applied to children with ASD.

## Methods

### Animals

B6;129-Nlgn3tm1Sud/J mice were obtained from Jackson Laboratories (Bar Harbor, Maine USA) and maintained to generation F9 on a Sv129/C57BL6 background. NL3^R451C^ and wild-type (WT) animals were derived by mating heterozygous females with NL3^R451C^ males, which produced 50:50 WT and NL3^R451C^ male offspring (Y/+ and Y/R451C) that were genotyped as previously described [9]. Experimental animals were weaned at 4 weeks of age and housed in groups of four per cage with food and water available *ad libitum*. The holding room was maintained on a 12:12-h light/dark cycle with lights on at 7.00 a.m. and at an ambient temperature of 20 ± 1 °C. All procedures were approved by the Florey Institute of Neuroscience and Mental Health Animal Ethics Committee. All behavioral testing was conducted in the light cycle, the animals were allowed at least 1 h to habituate to testing rooms, and the experimenter was blinded to genotype and exact sample sizes for each test are reported in figure legends. Four separate cohorts of mice were exposed to behavioral testing: cohort 1: repetitive object novel contact test (6 weeks of age) and olfactory discrimination (7 weeks of age); cohort 2: reciprocal juvenile social interaction test (3–4 weeks of age), social approach (9 weeks of age); cohort 3: light/dark arena test (7 weeks of age), elevated plus test (8 weeks of age); and cohort 4: resident-intruder test (10 weeks of age). 

### Repetitive object novel contact test

This test was designed to measure second-order repetitive behaviors (e.g., ritualistic patterns of behavior and insistence on sameness) and was based on a clinical test [26] and originally adapted for use on a BTBR mouse model of ASD [27]. On the day of testing, the WT and NL3^R451C^ mice were habituated to the experimental room for 30 min and to the testing arena for 20 min immediately prior to testing. The mice were placed in the 30 cm^2^ testing arena (lined with 1 cm of sawdust) with four distinct objects. One object occupied each corner of the arena during a 10-min trial. The four objects were as follows: a standard 6-sided dice (1.5 cm in length), a 20-sided dice (1.5 cm in diameter), a casino chip (2 cm in length), and a lego piece (2 cm in length) and placement was randomised to avoid any effect of circling or thigmotaxis behavior. Recorded video files were scored for object investigation by an observer blinded to genotype. Investigation was defined as clear facial or vibrissae contact and/or sniffing of the object. To assess for sequential patterns of investigation of the four objects, each object was allocated a number from one to four. The pattern of visitation of each individual mouse was then scored, and a record of investigation for each mouse was generated in a Microsoft Word document. For example, if the objects (designated 1–4) were visited in the designated order, the resulting sequence would be 1-2-3-4 in the word document. Subsequent visits were added contiguously to the visitation data string in the word document. The investigation data were subsequently searched using the ‘Find and Replace’ function in Microsoft Word for all possible four object visitation sequences. According to our criteria, a visitation sequence could include multiple visits to the same object, provided they were interrupted by a visit to another object. To determine if mice showed recurring visitation patterns, the total number of repetitions of identical four object visitation sequences was calculated for each mouse and then averaged by genotype.

### Grooming

The mice were placed in a clear Plexiglas cage (32 x 18 x 10 cm) lined with 1 cm of sawdust and allowed to habituate for 30 mins. They were then videoed for 10 mins and scored by a rater, blinded to genotype.

### Reciprocal juvenile social interaction

The mice aged 3–4 weeks were placed in a 30 cm^2^ cage with an unfamiliar mouse for 30 min. Equal representations of all possible pairs was achieved (WT/WT, WT/NL3, NL3/NL3). Testing sessions were recorded by a video camera mounted above the testing arena and multiple body point tracking software Cleversys LTD (Virginia, USA) was used to automatically measure time in social interaction.

### Social approach (three-chamber) test

The social approach test was performed as described [28]. In brief, after 10 min of habituation, a mouse was placed in the central chamber of a clear Plexiglas box (60 x 40 x 25 cm) divided into three interconnected chambers and was given the choice to interact with either an empty wire cup (located in one side of the chamber) or a similar wire cup with an unfamiliar 7–8-week-old C57BL/6J male mouse inside (located in the opposite chamber). Time in each chamber and interacting with each cup was measured by an observer blinded to genotype. The chambers were cleaned, and fresh bedding was added between trials. The position of the stranger mouse was randomised between the left and right side chambers to control for any chamber preference. The animals serving as strangers were habituated to placement under the wire cage for 5 min prior to the commencement of the test. Testing sessions were recorded by a video camera mounted above the testing arena, Cleversys LTD (Virginia, US) was used to automatically measure time in chambers, and time in direct social interaction was manually scored by a rater blinded to genotype.

### Olfactory discrimination test

This test was conducted in order to determine the ability of the mice to discriminate between and habituate to different odor types, e.g., social vs. non-social odors. The experimental room was cleared of all strong odors, and the mice were habituated to the room for a minimum of 1 h prior to testing. Concurrently, they were habituated to a clean cotton tip applicator placed in a weigh boat and inserted through the wire bars of the home cage. The testing arena consisted of a clear Plexiglas cage (32 × 18 × 10 cm) lined with 1 cm of sawdust. The odors were presented in sequential order: water, vanilla, almond, female urine, and male urine in accordance with a protocol previously described [29]. Vanilla and almond cooking essence were used, and urine was obtained from adult C57Bl/6J male and C57Bl/6J female mice in estrous. Each odor was presented for a period of 2 min, repeated over three trials with an inter-trial interval of 1 min. All odors were diluted in distilled water at a 1:4 dilutions factor and the investigator pipetted 10 μL of each odor onto a fresh cotton tip applicator, at the beginning of every trial. Recorded video files were scored for the amount of time the mouse spent sniffing the cotton tip applicator during each odor presentation by an observer blinded to genotype.

### Light-dark arena

Photo beam arenas (E63-10, TruScan, Coulbourn Instruments, Allentown, PA, USA) with a light-dark box insert placed over half of the arena were used to monitor the exploratory activity of the mice. The animals were placed in the light area facing into the dark area and allowed to enter. The light (750 lx) was then switched on, and the 10 min trial started.

### Elevated plus maze

The elevated plus maze consisted of two open (25 cm x 8 cm x 0.5 cm) and two closed (25 cm x 8 cm x 20 cm) arms emanating from a common central platform (8 cm x 8 cm) to form a plus shape and was elevated to 80 cm above floor level. The mice were habituated to the testing room for 1 h and then placed onto the central platform facing an open arm for a 6 min trial. The maze was thoroughly cleaned using 70 % ethanol between subjects, and scoring was performed using the Noldus Ethovision automated tracking system (version 3).

### Resident-intruder test

The male resident mice were isolated for 1 week, during which their home cages were not changed. Aggressive behaviors in 3-month-old mice were monitored during four 5 min test exposures to 8-week-old C57BL/6 male intruder mice conducted over 4 days as aggression is a highly variable behavior in mice [30, 31]. Mice were injected intraperitoneally with a non-sedative [32–37] dose of risperidone (0.05 mg/kg; Sigma) or saline 15 min prior to testing (Additional file 1: Figure S1). Testing was conducted by an experimenter blinded to genotype, and drug treatment and treatment group assignment was randomised ensuring sufficient spread of animals from different litters, housing boxes, and genotypes in each group. The trials were aborted if the experimenter observed tufts of hair being removed from either animal. Two NL3^R451C^ animals were excluded from testing on the first day due to extreme aggression towards the intruder. Latency to first attack, attack incidence, attack duration, tail rattles, and non-aggressive social interactions (sniffing, climbing, and grooming) were scored by a rater, blinded to genotype from video recordings of each test session.

### Statistical analyses

Where data distribution were normal and variance comparable (juvenile social interaction, adult social approach test, olfactory discrimination test, light-dark arena, elevated plus maze, repetitive object novel contact test, grooming), unpaired Student’s *t* test or ANOVAs with repeated measures where appropriate followed by Bonferroni post hoc tests were used as indicated. Aggression data was not normally distributed, and regression models were applied to estimate the effect size (Additional file 2: Figure S3). Two independent analyses were conducted in accordance with our hypotheses; (a) estimating the effect of the NL3R451C mutation on aggression and (b) estimating the effect of risperidone treatment on aggression in NL3^R451C^ mice. Due to absence of prior estimates of effect sizes, no power analysis was conducted. In both analyses, the animals were repeatedly tested over 4 days, thus these observations are correlated within a given animal. Hierarchical regression models were used to estimate the effect of the NL3R451C gene mutation on aggression and the effect of risperidone treatment on NL3^R451C^ mice. Both Bonferroni adjusted for multiple comparisons and unadjusted two-sided *p* values were reported together with appropriate effect size estimates and 95 % confidence intervals (95 % CI) to indicate the precision. The latency data describes the time to an attack and may be censored (e.g., when an animal does not attack during the 300-s observation period). Survival analysis, in particular the Cox regression model, is appropriate for analysis of these data [38]. A hierarchical Cox regression model that accounts for the effect of multiple test days was used to estimate the effect size, measured as the hazard ratio of the first attack occurring at any time over the 300-s observation period. Attack incidence is a count variable, and a hierarchical Poisson regression model was used to estimate the effect size, measured as the ratio of expected number of attacks. Although the duration of social interaction data violated the normality assumption, a hierarchical random effects generalised least squares regression model did not violate the assumptions of model fit. Both Bonferroni adjusted for multiple comparisons and unadjusted two-sided *p* values were reported together with appropriate effect size estimates and (95 % CI) to indicate the precision. Results for aggression data are graphically presented as a box and whiskers plot showing the 25th to the 75th percentile and the minimum to maximum of the data range and the median was shown by a line. Statistical analyses were performed with STATA and SPSS software.

## Results

### NL3^R451C^ mice exhibit repetitive behavior in an object exploration task

**Fig. 1:**
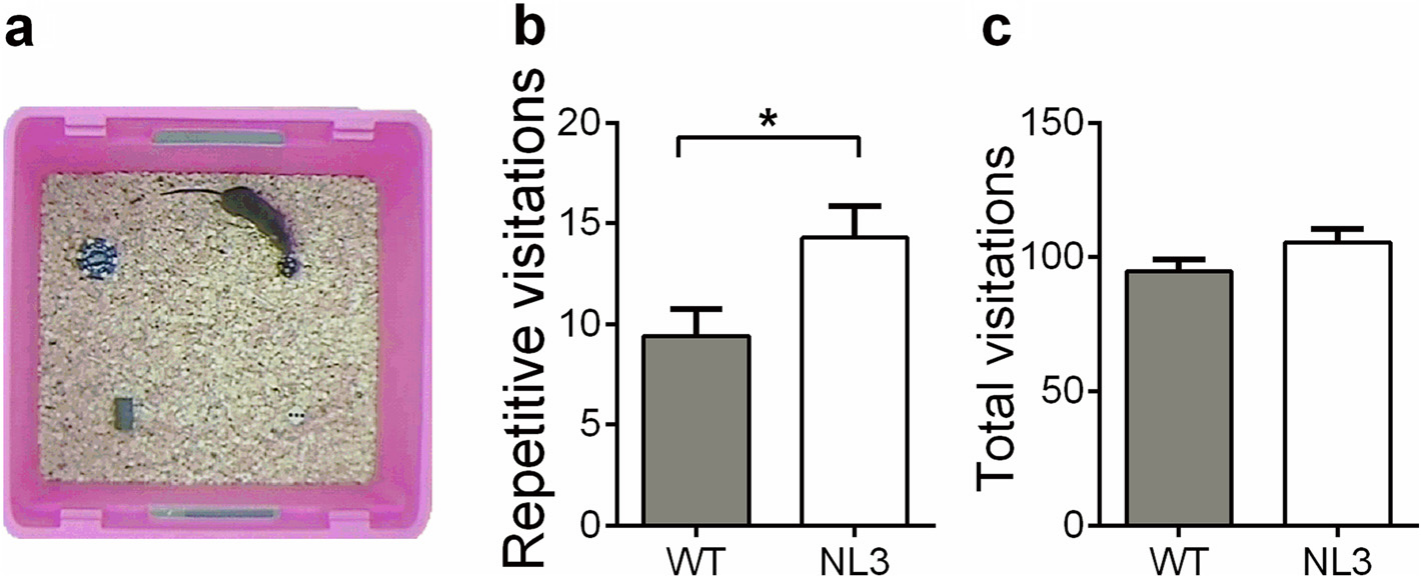
NL3^R451C^ mice display increased repetitive behavior in the repetitive novel object contact test. **a** The arena contained four clearly distinguishable objects, one located in each corner, and the repetition of visitation patterns was recorded as a measure of repetitive/ritualistic behavior. **b** The NL3^R451C^ mice showed increased repetition of identical object sequences of visitation (four objects) compared to the WT. A four-object visitation sequence could include multiple visits to the same object, provided they were interrupted by a visit to another object. **c** The NL3^R451C^ and WT mice made a comparable number of total visits to novel objects. Values are displayed as mean ± SEM. *Asterisks* represent a statistically significant difference between the indicated groups. *p < 0.05; *n* = 21 mice in each group

The repetitive novel object contact test involved placing four clearly distinguishable objects (one in each corner) in a neutral arena, and repetition of visitation patterns were recorded as a measure of repetitive/ritualistic behavior (Fig. 1a). The NL3^R451C^ mice repeated sequential patterns of investigation consisting of four objects more often than the WT mice (Fig. 1b; *t*_45_ = −2.35, *p* = 0.024). The total number of object visitations was assessed as a measure of general exploration and hyperactivity and were comparable between the NL3^R451C^ and WT mice (Fig. 1c). Grooming was assessed as a measure of stereotypy, and no differences were uncovered between the NL3^R451C^ mice and WT mice (Additional file 2: Figure S3).

### Juvenile social interaction was impaired in NL3^R451C^ mice, but adult mice show comparable sociability to WT mice

**Fig. 2:**
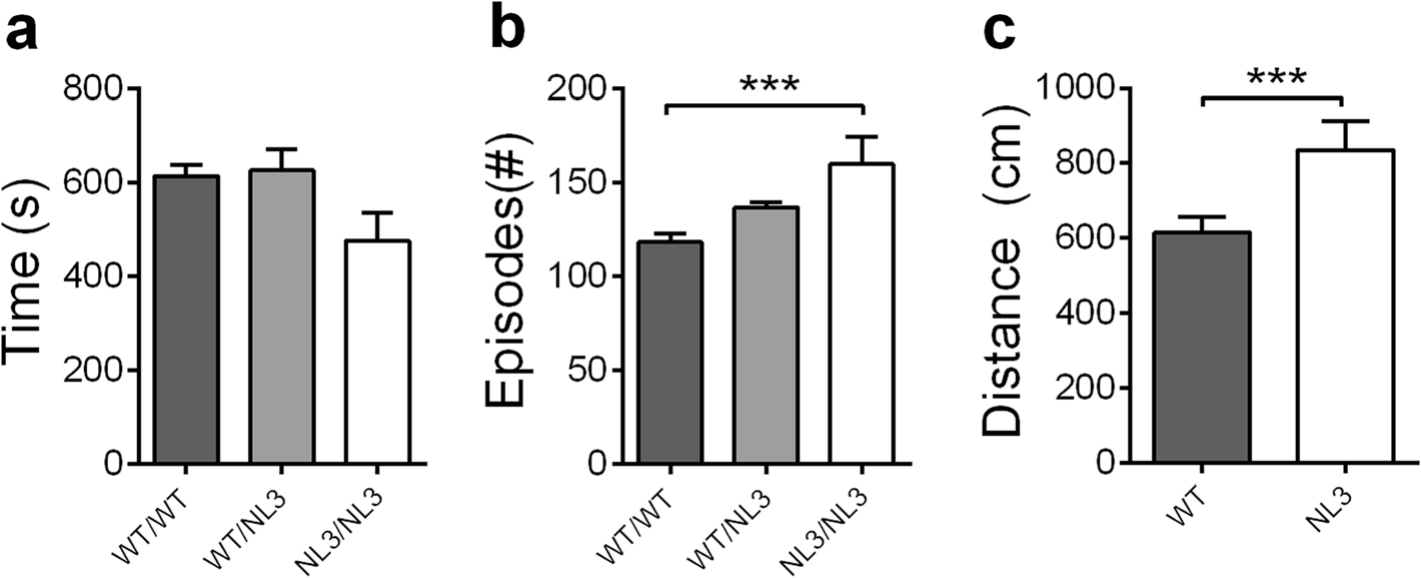
Juvenile NL3^R451C^ mice display abnormal social interaction in a free interaction arena. **a** A trend for the juvenile NL3^R451C^ mice to spend less time interacting with novel genotype-matched (NL3/NL3; *n* = 10) mice compared to mixed genotype pairs (WT/NL3; *n* = 14) and WT mice (WT/WT; *n* = 15) was seen. **b** The NL3^R451C^ mice engage in more episodes of social interactions and **c** cover a greater distance in the arena during the assay compared to the WT mice. Values are displayed as mean ± SEM. *Asterisks* represent a statistically significant difference between the indicated groups. ***p < 0.001

Juvenile social interaction was assessed in mice aged 3–4 weeks paired with age- and sex-matched same or opposite genotype mice. One-way ANOVA revealed a significant main effect of the group (Fig. 2a; *F*_2,37_ = 3.54, *p* = 0.039); however, post hoc comparisons were not significant. The NL3^R451C^ mice paired with the same genotype mice showed a trend for reduced social interaction compared to the WT/WT pairs (*p* = 0.08) and compared to the NL3^R451C^ mice paired with WT mice (*p* = 0.058). The NL3^R451C^ mice in same genotype pairs were more likely to make social contact compared to the WT/WT pairs (Fig. 2b; F_2, 37_ = 7.44, *p* = 0.002; WT/WT vs. NL3/NL3, *p* < 0.001). This increase in episodes of social interaction but not in duration could be due to the NL3^R451C^ mice exhibiting hyperactivity in the arena. The total distance covered by the NL3^R451C^ mice was significantly greater when compared to the WT (Fig. 2c; *t*_17_ = −2.7, *p* = 0.015).

**Fig. 3:**
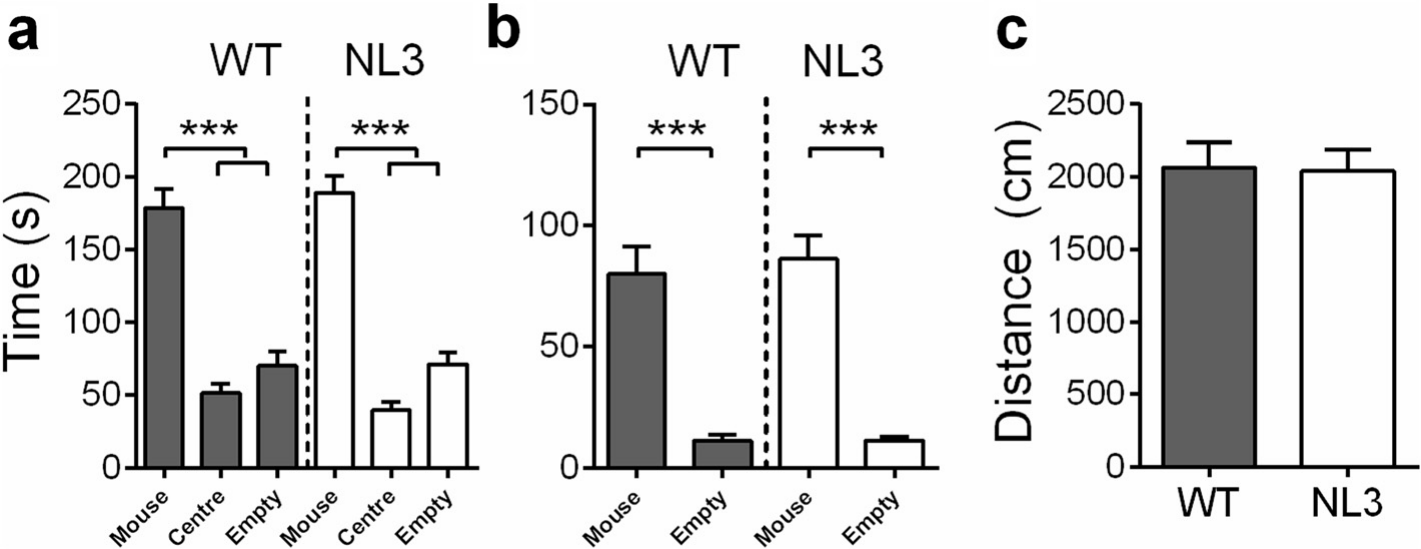
**a** In the three-chamber interaction task, adult WT and NL3^R451C^ mice demonstrated preference for the chamber containing the novel C57Bl6 mouse and also for **b** interacting directly with this caged mouse vs. the empty cage. **c** No difference in distance covered was seen between genotypes. Values are displayed as mean ± SEM. *Asterisks* represent a statistically significant difference between the indicated groups. ***p < 0.001; *n* = 10 mice in each group

We investigated this change in social behavior in an independent experiment with the widely used social approach (three-chamber) test performed in adult mice at 9 weeks. In this test, mice are given the choice to interact with either an empty cage or a similar cage containing an unfamiliar stimulus mouse, matched for age and sex. The amount of time in the chamber containing the mouse and also interacting with the cage containing the mouse is compared as a measure of sociability. Normal sociability is defined as spending significantly more time interacting with the cage containing the mouse than with the empty cage [39, 40]. One-way ANOVA with repeated measures (chamber) revealed a significant effect of chamber (Fig. 3a; main effect of chamber: *F*_2,34_ = 79.28; *p* < 0.001). Both the WT and NL3^R451C^ mice showed normal sociability, demonstrating preference for the chamber containing the stranger mouse (mouse vs. center, empty *p* < 0.001). Normal sociability in both genotypes was also reflected in directed interactions with the mouse vs. the empty cage (Fig. 3b; one-way ANOVA main effect of cage: *F*_1,17_ = 89.85; *p* < 0.001, mouse vs. empty *p* < 0.001). WT and NL3^R451C^ mice cover comparable differences during the social interaction trial (Fig. 3c).

### NL3^R451C^ mice display normal olfactory habituation and dishabituation to social and non-social odors

**Fig. 4:**
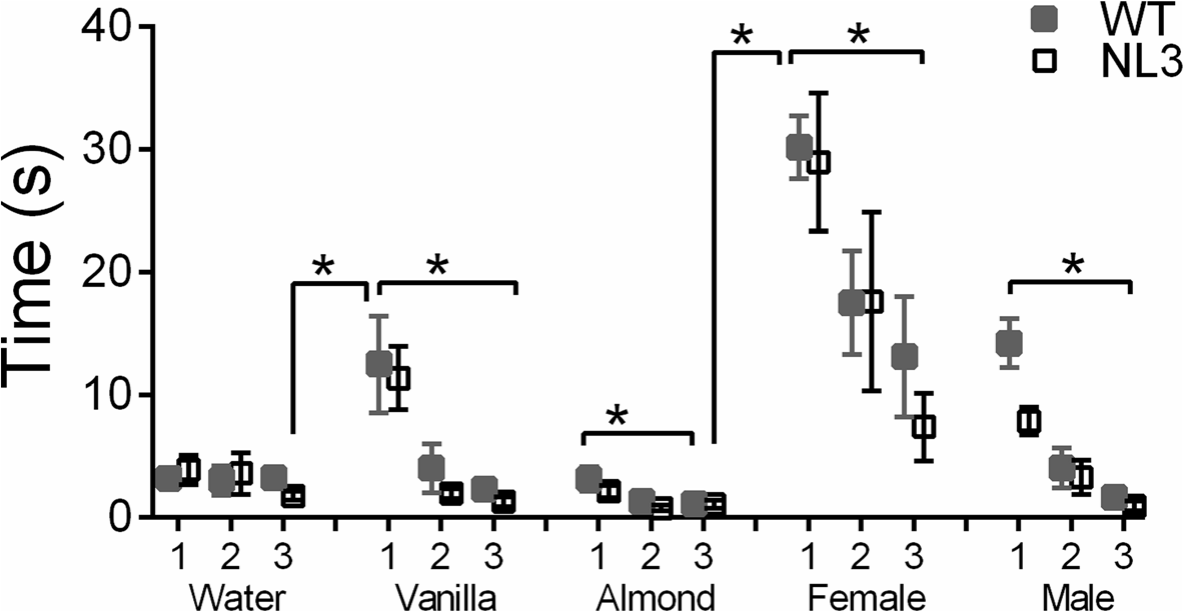
NL3^R451C^ mice display normal olfactory habituation and dishabituation. There were no significant differences in the habituation and dishabituation patterns displayed by the WT (*n* = 27) and NL3^R451C^ mice (*n* = 21). Both genotypes exhibited significant dishabituation of water to vanilla and female urine and habituation to vanilla, almond, female urine, and male urine. Values are displayed as mean ± SEM. *Asterisks* represent a statistically significant difference between indicated groups. *p < 0.05

Sniffing time in both WT and NL3^R451C^ mice was influenced by both the type of odorant presented (Fig. 4; *F*_4,180_ = 30.74, *p* < 0.001) and whether it was the first, second, or third presentations of that odorant with the first presentation of each odorant eliciting greater sniffing behavior (*F*_2,90_ = 51.71, *p* < 0.001). There was also a significant interaction of sniffing time and order of odorant presentation (*F*_8,360_ = 8.36, *p* < 0.001) as indicated by repeated measures ANOVA. Both the WT and NL3^R451C^ mice exhibited significantly longer durations sniffing female urine on first presentation when compared to all other odors. The NL3^R451C^ and WT mice exhibited similar preferences for odorants, and there was no interaction between odorant and genotype. All mice showed an aversive reaction to almond; however, the duration of time spent sniffing water, vanilla, and male urine did not differ significantly.

### NL3^R451C^ mice show normal anxiety levels

The NL3^R451C^ mice show no difference in the percentage of time they spend in the light side of the light-dark arena compared to the WT mice (Additional file 3: Figure S2a). Similarly, the NL3^R451C^ and WT mice show comparable time in the open and closed arms of the elevated plus maze (Additional file 3: Figure S2b).

### Heightened aggression in NL3^R451C^ mutant mice is reversed with risperidone treatment

The NL3^R451C^ and WT mice were assessed for aggressive behavior utilising the resident-intruder test, whereby a juvenile intruder mouse is introduced to the home cage of a test mouse. This paradigm mimics innate social behavior displayed by resident males to exclude other breeding males from their home territory and their mates [23]. Abnormal aggression is characterised by brief latencies to attack and prolonged periods of aggression following the introduction of an intruder mouse [24].

**Fig. 5:**
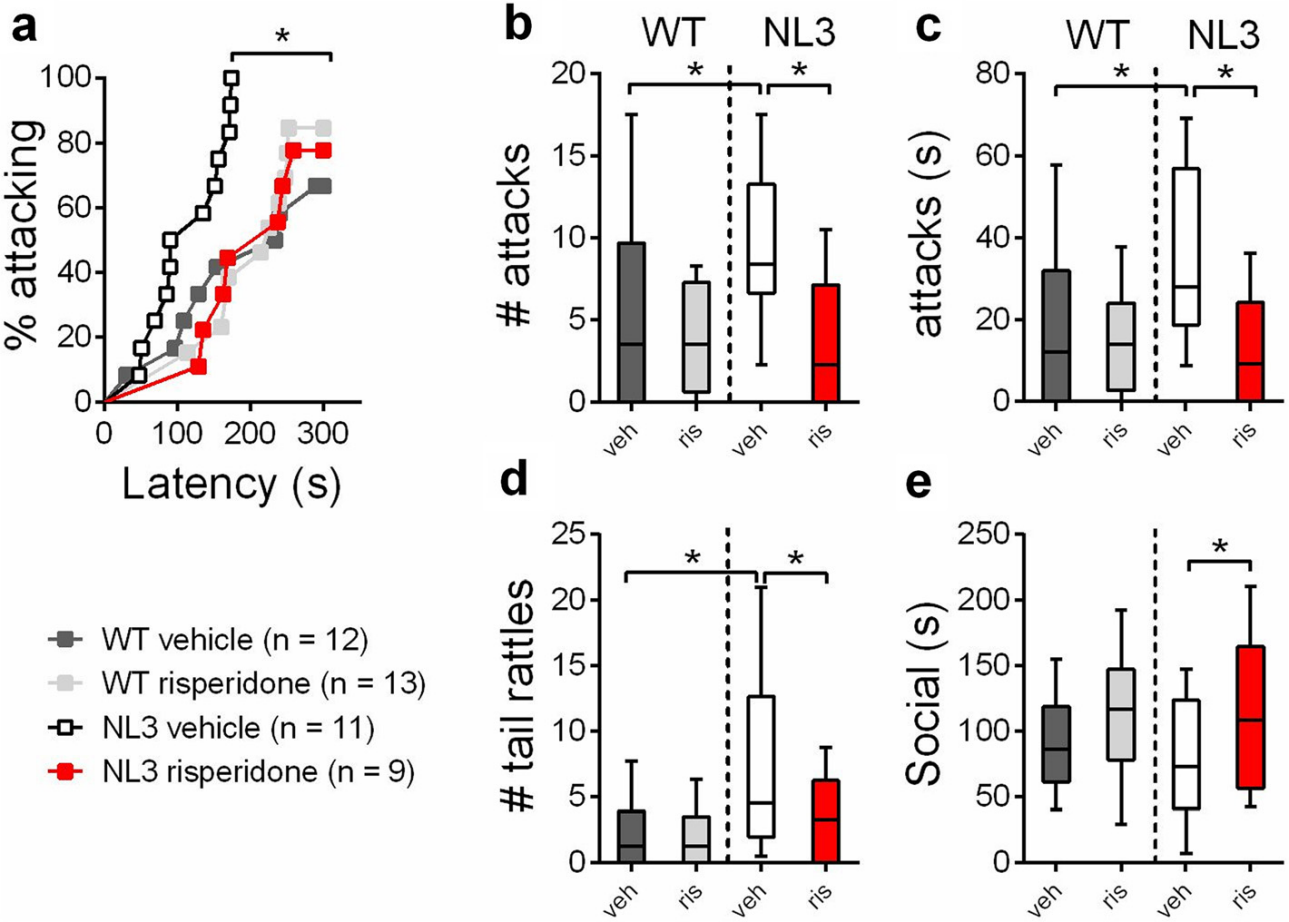
NL3^R451C^ mice display heightened aggression which is rescued by risperidone. **a** Vehicle-treated NL3^R451C^ mice showed an increased risk of attacking compared to WT mice, which was reversed with risperidone. In the WT mice, the risk of an attack was unchanged following risperidone treatment. The NL3^R451C^ mice attacked **b** more often , **c** for longer and **d** displayed more tail rattles compared to the WT mice. All measures of aggression were also reduced by risperidone. **e** Risperidone increased the duration of agonistic social interactions in the NL3^R451C^ mice compared to the vehicle-treated mice. Values for **a**–**e** are an average of four tests, and data in **b**–**e** are displayed as *box plots* with medians plus the 25^th^ and 75^th^ percentiles. Whiskers represent the minimum and maximum values. Asterisks represent a statistically significant difference between indicated groups. *p < 0.05

When confronted by an intruder, the resident vehicle-treated NL3^R451C^ mice were 7.8-fold more likely to initiate a first attack compared to the WT mice (Fig. 5a; hazard ratio = 7.84, *p* = 0.003; 95 % CI 2.04, 30.22; for all statistical details, refer to Additional file 4: Table S1). The NL3^R451C^ mice also attacked more often (Fig. 5b; ratio of expected number of attacks = 24.84; *p* < 0.001; 95 % CI 7.45, 82.84) and for longer periods of time than the WT littermates (Fig. 5c; difference in mean duration = 28; *p* < 0.001; 95 % CI 14, 41). Furthermore, the NL3^R451C^ mice displayed significantly more tail rattles (a territorial behavior initiated in response to a threat) than the WT animals (Fig. 5d; ratio of expected number of tail rattles = 20.92; *p* < 0.001; 95 % CI 6.40, 68.39).

An important goal in developing mouse models of neuropsychiatric diseases is testing therapeutic treatments. Risperidone was the first drug approved by the US Food and Drug Administration (FDA) for symptomatic treatment of ASD, alleviating aggression and repetitive, self-injurious behavior [41, 42]. The NL3^R451C^ mice and their WT littermates were treated with risperidone or vehicle and were tested for improvement in aggressive behavior. There were no significant changes in open field activity in the risperidone-treated NL3^R451C^ mice, indicating that the dose used was not sedating (Additional file 1: Figure S1). We found that risperidone significantly reduced aggression in the NL3^R451C^ mice (Fig. 5a–e). Risperidone treatment reduced the risk of attack in the NL3^R451C^ mice and had no effect on the WT mice (Fig. 5a; hazard ratio = 0.22, 95 % CI 0.10, 0.55; *p* < 0.001). The number of attacks and attack duration were both reduced by risperidone (Fig. 5b; ratio of expected number of attacks = 0.41; *p* < 0.001; 95 % CI 0.27, 0.64; Fig. 5c; difference in mean duration = −22; *p* < 0.001; 95 % CI −35, −8). The risperidone-treated NL3^R451C^ mice displayed less tail rattles towards intruders compared to the vehicle-treated mutants (Fig. 5d; ratio of expected number of tail rattles = 0.47; *p* = 0.023; 95 % CI 0.25, 0.90). While the NL3^R451C^ mice did not show a reduction in agonistic social behavior, risperidone increased the time that mutants spent engaging in this behavior (Fig. 5e; difference in mean duration = 58 s; *p* < 0.001; 95 % CI 24, 92).

## Discussion

The development of mouse models of ASD is crucial to study the disorder at molecular and cellular levels, to gain insight into disease mechanisms, and test potential pharmacological interventions. Here, we have shown that risperidone efficiently reduces aggression in the NL3^R451C^ mouse, while having no effect on the WT behavior or on locomotor activity levels. This is the first demonstration of risperidone rescuing aggression in mice with an ASD-associated genetic mutation and demonstrates predictive validity in this model. The NL3^R451C^ mice exhibit normal anxiety levels and olfactory discrimination indicating that abnormal aggressive behavior is not due to altered anxiety or olfaction. Furthermore, while we uncover subtle differences in social behavior in the juvenile NL3^R451C^ mice, as adults, mutants show similar sociability to the WT littermates. Using a novel assay of repetitive and restrictive behavior, we show that the NL3^R451C^ mice exhibit stereotypical object exploration, further confirming the face validity of the NL3^R451C^ model in the context of ASD.

Previous characterisations of NL3^R451C^ mouse behavior have focused on potential deficits in social interactions [9, 13–15], but less attention has been given to the analysis of repetitive and restrictive movements and routines. In order to assess this domain in the NL3^R451C^ mice, we employed a behavioral assay based on findings from a clinical trial demonstrating that children with ASD tended to play with toys in a particular order [26]. Upon exposure to four objects, we observed that the NL3^R451C^ mice repeated particular patterns of visitation more frequently than the WT littermates. Compared to the initial study by Pearson et al., utilising this task, we report a higher frequency of four sequence patterns, despite time and total visitations being similar. This discrepancy is most likely due to the fact that both studies defined a visitation sequence as one that could include multiple visits to the same object, provided they were interrupted by a visit to another object. It is possible therefore that the mice in the study by Pearson et al. made more consecutive visits to the same object, which would lower the frequency of four-object sequences recorded. The emphasis of this task is primarily on the repetition of these sequences of investigation rather than the specific order of objects. If mice were retested, it is likely that each NL3^R451C^ mouse would generate a different pattern of investigation, but that the object exploration would be repeated to a higher degree than WT mice. The exact order of object exploration would depend upon the time between the initial and subsequent tests and the ability of mice to recall their previous visitation pattern. Given the novelty of this test, this point has not been addressed in the behavioral literature and is one that we may explore in the future.

It has also been recently shown that NL3^R451C^ mutants show enhanced acquisition of repetitive motor routines on the rotarod [43]. This repetitive phenotype was recapitulated by the conditional deletion of NL3 in adult mice and was shown to be exclusively mediated by a reduction of synaptic inhibition onto ventral striatum dopamine D1 medium spiny neurons. While the neural mechanisms underlying object exploration are likely to differ from those governing motor habit formation, it is likely that the NL3^R451C^ mutation could disrupt striatal circuits to shape a broad range of repetitive and stereotypic behaviors associated with ASD. This has been demonstrated by volumetric MRI studies that have shown smaller striatal volume in the NL3^R451C^ mice [44, 45]. Furthermore, the disruption of striatal circuitry demonstrated in the NL3^R451C^ mouse model is in keeping with striatal structural and functional alterations in ASD patients [46–48].

NL3^R451C^ mice have been assessed for sociability in a number of studies using the three-chamber social interaction arena assay; a task that tests for preference for an empty cage vs. caged novel mouse simultaneously. We show that the NL3^R451C^ mice show a strong preference for the novel mouse over the empty cage, indicating no difference in sociability compared to the WT animals. While the first investigation of adult social behavior in NL3^R451C^ mice on the C57Bl6-SV129 hybrid background showed decreased time in the chamber containing the novel mouse compared to WT mice, mutant mice still displayed a preference for the novel mouse (more time with novel mouse than with novel object) [9]. There was no significant genotype difference when reciprocal social interactions were measured. Other behavioral assays conducted in that study [9] do not represent standard measures of social choice. Using the three-chamber arena, the same group later characterised social interaction in the NL3^R451C^ mice on a pure C57Bl6 background and reported a strong preference for the novel mouse over the novel object in both the WT and mutant mice, indicating no decrease in sociability [11]. When the object was replaced with another mouse, however, the NL3^R451C^ mice were shown to have a deficit in social recognition. Our results are in agreement with Crawley et al. assessing sociability in NL3^R451C^ mice on the C57Bl6 background [15]. This study also conducted the three-chamber social interaction assay on a cohort of mice naïve of testing, removing any potential confound of prior experience. Two research groups have shown deficits in social interaction in this mutant on the same C57Bl6-SV129 hybrid background and on a pure 129S2/SvPasCrl background [13, 45]. Discrepancies in social behavior assessed in the three-chamber arena could be attributable to differences in genetic background strain, differences in methodology (e.g., lighting conditions and previous behavioral testing experience) or even as a result of testing in different laboratories. We assessed juvenile social interaction in the WT and NL3^R451C^ mice at 4 weeks of age and found no difference in total time spent interacting with the novel mouse between genotypes, in line with another study investigating juvenile interaction in NL3^R451C^ mice [13]. Although the increased frequency of interaction seen in the mutants in the current study contrasts with one report that juvenile NL3^R451C^ mice show no difference in this parameter [15], our present finding is likely a result of hyperactivity shown by the NL3^R451C^ mice during the 30-min testing period. No aggression was noted during juvenile social interaction testing, suggesting that the heightened aggression expressed in the adult NL3^R451C^ mouse could be territorial in nature.

We have demonstrated that heightened aggression is a robust phenotype in the NL3^R451C^ mouse model of ASD. While we assessed aggression specifically towards a novel juvenile mouse following a period of isolation, a lower incidence of aggression was noted in the home cages of the animals. This observation has implications for interpretation of adult behavior in studies utilising this mutant, as repeated exposure to aggression can lead to stress, alter baseline behavior in the WT mice and impact on health [49]. Furthermore, this is the first demonstration that risperidone can ameliorate aggression in a mouse model of ASD. How risperidone decreases aggression in NL3^R451C^ mice is not known. Risperidone has a broad antagonist receptor profile with greatest affinity for serotonin (5-hydroxy-tryptamine (5-HT)) 2A receptors, followed by 5-HT1B, 5-HT7, and dopamine D2 receptors [50]. Both antagonists acting at 5-HT2A [51, 52] and D2 receptors [53, 54] reduce aggressive behaviors in mice, suggesting that these specific receptor candidates may mediate the effects of risperidone in NL3^R451C^ mice; however, this requires further examination.

## Conclusions

The serious negative outcomes associated with behavioral impairments in ASD highlight the inherent importance of understanding the underlying neurobiological mechanisms, with the aim of developing more effective treatments. We have shown novel behavioral phenotypes of repetitive and restrictive object exploration and aggression in the NL3^R451C^ mice, strengthening the face validity of the mouse model. Social impairments are considered an essential readout when validating ASD animal models, and while our findings showed normal interaction in the three-chamber assay, the expression of heightened aggression in the NL3^R451C^ mice indicates that abnormal social behavior is indeed present in this mutant. Our demonstration that risperidone reduced aggression in the NL3^R451C^ mice is the first of its type in ASD research and is in line with clinical treatment of aggression in ASD. The NL3^R451C^ mouse model of ASD demonstrates construct validity, face validity, and now strong predictive validity and represents a powerful tool to explore pathogenesis and the development of new treatments for ASD.

## Additional files

Additional file 1: Figure S1. Risperidone (0.05 mg/kg) treatment did not alter locomotor activity in NL3^R451C^ mice. NL3^R451C^ mice treated with risperidone (*n* = 6) showed no difference in distance travelled compared to vehicle-treated controls (*n* = 4) as assessed in Truscan (Coulbourn Instruments) locomotor activity arenas (two-way repeated measures ANOVA, F1,9 = 0.1; *p* = 0.75). Arrow denotes time of drug or saline treatment. Values are displayed as mean + SEM. Additional file 2: Figure S3. No differences between grooming behavior were observed between NL3^R451C^ (*n* = 12) and WT mice (*n* = 14). Values are displayed as mean ± SEM. Additional file 3: Figure S2. NL3^R451C^ mice show no differences in measures of anxiety. (a) WT and NL3^R451C^ mice spent comparable time in the light side of the light-dark arena. (b) Both WT and NL3^R451C^ mice showed a preference for the closed arm in the elevated plus maze and no differences in time spent in either closed or open arms. Values are displayed as mean ± SEM. *n* = 10 mice in each group. Additional file 4: Table S1. The effect of risperidone treatment on the risk of aggressive behavior in WT and NL3^R451C^ mice. Hierarchical Cox regression was used to estimate the effect size (ES) of latency to attack, measured as the hazard ratio of the first attack occurring at any time over the 300-s observation period. Number of attacks and tail rattles were analyzed with hierarchical Poisson regression models, and effect sizes are listed as the ratio of expected number of attacks or tail rattles. Hierarchical random effects generalised least squares regression models were used to estimate the mean difference for duration of attacks and social interaction. Effect size, 95 % confidence intervals (CI), and *p* values for each parameter are shown for interaction (genotype vs. treatment), genotype (WT vs. NL3) and treatment (vehicle vs. risperidone) comparisons. All analyses were adjusted for the effect of day.

## Abbreviations

5-HT: 5-hydroxy-tryptamine (serotonin)
ASD: autism spectrum disorders
D1: dopamine D1 receptors
D2: dopamine D2 receptors
FDA: US Food and Drug Administration
NL3: neuroligin-3
R451C: arginine to cysteine residue 451 substitution
WT: wild-type
