# Supplementary figures and images for: A neuroligin-3 mutation implicated in autism causes abnormal aggression and increases repetitive behavior in mice
Source: Mol Autism. 2015 Nov 14;6:62. doi: 10.1186/s13229-015-0055-7 (PMC4650404; doi:10.1186/s13229-015-0055-7)

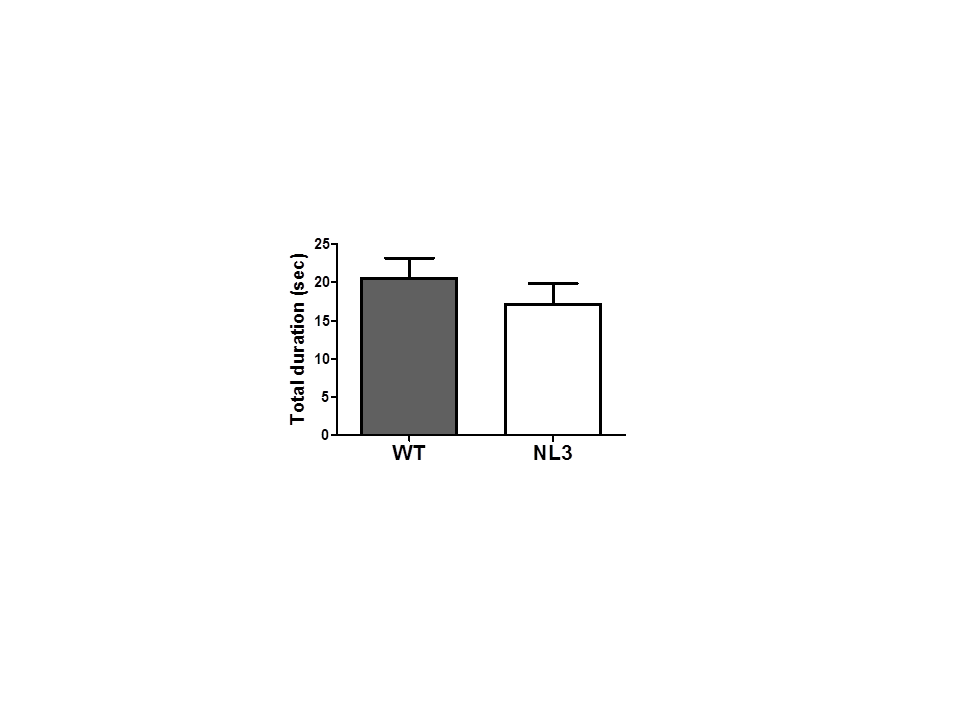

Supplement: Additional file 2: Figure S3. — No differences between grooming behavior were observed between NL3R451C (n = 12) and WT mice (n = 14). Values are displayed as mean ± SEM. [file 13229_2015_55_MOESM2_ESM.tif]

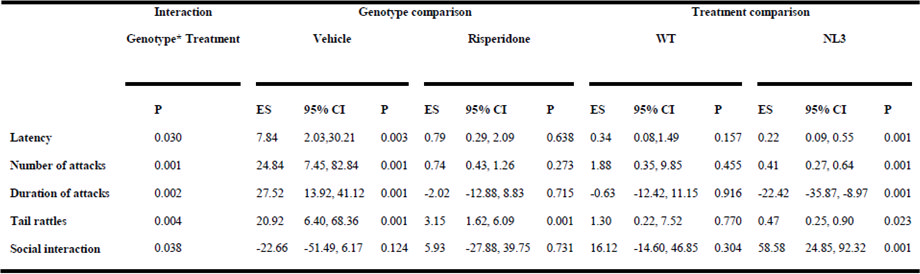

Supplement: Additional file 4: Table S1. — The effect of risperidone treatment on the risk of aggressive behavior in WT and NL3R451C mice. Hierarchical Cox regression was used to estimate the effect size (ES) of latency to attack, measured as the hazard ratio of the first attack occurring at any time over the 300-s observation period. Number of attacks and tail rattles were analyzed with hierarchical Poisson regression models, and effect sizes are listed as the ratio of expected number of attacks or tail rattles. Hierarchical random effects generalised least squares regression models were used to estimate the mean difference for duration of attacks and social interaction. Effect size, 95 % confidence intervals (CI), and p values for each parameter are shown for interaction (genotype vs. treatment), genotype (WT vs. NL3) and treatment (vehicle vs. risperidone) comparisons. All analyses were adjusted for the effect of day. [file 13229_2015_55_MOESM4_ESM.tif]
